# Somatic symptom severity association with healthcare utilization and costs in surgical inpatients with an episode of abdominal pain

**DOI:** 10.1093/bjsopen/zrac046

**Published:** 2022-07-07

**Authors:** Melissa Stieler, Peter Pockney, Cassidy Campbell, Vaisnavi Thirugnanasundralingam, Lachlan Gan, Matthew J Spittal, Gregory Carter

**Affiliations:** College of Health, Medicine and Wellbeing, School of Medicine and Health Sciences, University of Newcastle, Callaghan, New South Wales, Australia; Department of Surgery, John Hunter Hospital, Newcastle, New South Wales, Australia; College of Health, Medicine and Wellbeing, School of Medicine and Health Sciences, University of Newcastle, Callaghan, New South Wales, Australia; Department of Surgery, John Hunter Hospital, Newcastle, New South Wales, Australia; Department of Surgery, John Hunter Hospital, Newcastle, New South Wales, Australia; Department of Surgery, John Hunter Hospital, Newcastle, New South Wales, Australia; Melbourne School of Population and Global Health, The University of Melbourne, Victoria, Australia; College of Health, Medicine and Wellbeing, School of Medicine and Health Sciences, University of Newcastle, Callaghan, New South Wales, Australia; Department of Consultation-Liaison Psychiatry, Calvary Mater Newcastle, Waratah, New South Wales, Australia

## Abstract

**Background:**

Somatic syndromes are present in 30 per cent of primary healthcare populations and are associated with increased health service use and health costs. Less is known about secondary care surgical inpatient populations.

**Methods:**

This was a prospective longitudinal cohort study (*n* = 465) of consecutive adult admissions with an episode of non-traumatic abdominal pain, to the Acute General Surgical Unit at a tertiary hospital in New South Wales, Australia. Somatic symptom severity (SSS) was dichotomized using the Patient Health Questionnaire (PHQ)-15 with a cut-off point of 10 or higher (medium–high SSS) and compared pre-admission and during admission. Total healthcare utilization and direct costs were stratified by a PHQ-15 score of 10 or higher. Linear regression was used to examine differences in costs, and a multivariable linear regression was used to examine the relationship of PHQ-15 scores of 10 or higher to total costs, reported as mean total costs of care and percentage difference (95 per cent confidence intervals).

**Results:**

Fifty-two per cent (*n* = 242) of participants had a medium–high SSS with greater pre-admission and admission interval health service costs. Mean total direct costs of care were 25 per cent (95 per cent c.i. 8 to 44 per cent) higher in the PHQ-15 score of 10 or higher group: mean difference €1401.93 (95 per cent c.i. €512.19 to €2273.67). The multivariable model showed a significant association of PHQ-15 scores of 10 or higher (2.1 per cent; 0.2–4.1 per cent greater for each one-point increase in score) with total hospital costs, although the strongest contributions to cost were older age, operative management, and lower socioeconomic level. There was a linear relationship between PHQ scores and total healthcare costs.

**Conclusions:**

Medium to high levels of somatic symptoms are common in surgical inpatients with abdominal pain and are independently associated with greater healthcare utilization.

## Teaser

Somatic syndromes are common psychiatric presentations in primary and secondary care, characterized by illness anxiety surrounding physical symptoms. In an inpatient surgical population presenting with abdominal pain, somatic symptom severity was associated with higher inpatient and outpatient healthcare expenditure, and increased duration of hospital stay.

## Introduction

Abdominal pain is a common presentation to the emergency department (ED), accounting for 7 per cent of all ED presentations^1^, with 18 per cent requiring admission^2^. Patients with abdominal pain are most frequently admitted under surgeons for further investigation and treatment or discharged from ED without admission. Although managed by surgeons, more than half of abdominal pain admission patients are managed with non-operative care^3^.

Somatic syndromes are marked by excessive thoughts, feelings or behaviours related to somatic symptoms and associated health concerns. This is manifested as persistent anxiety about health that cause significant disruption to daily life. The latest iteration of the Diagnostic Statistical Manual of Mental Disorders, the DSM-5, has highlighted the need to recognize somatic symptoms in patients who also have underlying medical disease^4^. Somatic symptoms are varied and commonly include neurological, musculoskeletal, and gastrointestinal systems^5^. Varied symptomology leads patients to present to many different specialists to find a cause for and resolution of their symptoms. Patients suffering from somatoform syndromes have high rates of co-morbid anxiety and depression and have substantial functional impairment^6^.

Somatoform syndromes have been associated with 1.8 times the rate of outpatient healthcare visits (3.18 *versus* 5.82) in one general population study^7^, 1.5 times as many primary care visits, and 3 times as many hospitalizations, major outpatient procedures and ED visits in a primary care population^8^. The total annual medical costs for a ‘somatizer’ was 2.3 times that for a ‘non-somatizer’ (€1740 *versus* €4003). Extrapolating this to the US healthcare system at large, the authors estimated the annual incremental cost of somatization to be €161 billion^8^.

Despite the high prevalence, and associated healthcare cost burden in primary care populations, little is known about the prevalence of somatoform syndromes in surgical inpatients, or the associated healthcare utilization and costs. The aim of this study was to estimate the healthcare utilization and costs of surgical inpatients with abdominal pain in a secondary care centre, and compare service use and costs for participants with medium to high somatic symptom severity and low symptom severity.

## Methods

### Study design and population

This study was conducted at a 694-bed tertiary hospital in New South Wales, Australia (John Hunter Hospital) a prospective cohort of consecutive adult (aged 18 years or older) patients admitted to the Acute General Surgical Unit (AGSU) with any form of non-traumatic abdominal pain.

Exclusion criteria included: age less than 18 years, inability or unwillingness (such as developmental delay or being significantly unwell and requiring ICU admission) to provide informed consent; or any adult patient with abdominal pain initially admitted to other services such as gynaecology, gastroenterology or general medicine. Only the index admission in the study interval was used for analysis. Recruitment was intended to continue until 1000 participants were included; however, the recruitment process was terminated prematurely due to compulsory, hospital-wide suspension of most non-COVID-related clinical research activity after the onset of the COVID-19 pandemic in March 2020.

### Ethics approval

This study was approved by the Hunter New England Human Research Ethics Committee Regis SSA reference no. 2018STE00509 on 20 May 2019.

### Study procedures

After admission to the surgical ward, eligible patients were initially approached by a member of the clinical service and if interested in participation, the study and recruitment process was undertaken by either a surgical registrar or resident, who were members of the research team. Participants provided written informed consent for participation in the study, including the collection of individual data from the clinical record and the study instruments. Consenting participants were provided with an envelope containing the study questionnaires. The participants were encouraged to complete the questionnaires on their own if able, and if unable due to visual impairment, poor literacy or other reasons, participants could be assisted by a research team member or nursing staff. Upon completion of the questionnaires, participants were encouraged to insert and seal the materials into a provided envelope and return it to research staff before discharge, or to mail the envelope to the research team after discharge. If participants wished to withdraw consent, they could do so at any point during their admission and the sealed envelope would be returned to the patient. The envelope was not opened during the index admission to maintain blindness of the research and surgical treatment team.

### Measures

#### PHQ-15 questionnaire

The Patient Health Questionnaire (PHQ)-15 is an instrument designed to assist physicians to efficiently and accurately screen for somatoform syndromes and monitor somatic symptom severity. It consists of 15 questions that enquire about the most prevalent somatic symptoms^5^, including four pain symptoms, two gastrointestinal symptoms, one sexual symptom and one pseudo-neurological symptom^9^. It has been validated as a screener for Diagnostic Statistical Manual (DSM)-IV somatization disorder^9^, DSM-5 somatic symptom disorder^10^, and somatic symptom severity in medical inpatients^11^. The questionnaire is scored 0–30, with scores of 5, 10 and 15 representing cut-off points for low, medium and high somatic symptom levels respectively^12^. The PHQ-15 score cut-off point of 10 or higher is the most frequently used to report prevalence of ‘somatization syndromes’^13^ as well as a validated screening instrument of ‘somatization’^14^ and in diagnostic accuracy studies to identify DSM-IV somatoform diagnoses, against a structured diagnostic interview^15,16^.

The PHQ-15 score cut-off point of 10 or higher has optimal sensitivity and specificity for identifying ‘somatization disorder’ (80.2 per cent and 58.5 per cent respectively)^12^. It can be used without further inquiry into the patient’s medical co-morbidity, in the context of examining healthcare utilization by people who score above and below the threshold levels, as physical co-morbidity does not alter utilization significantly in primary care populations^8^.

The PHQ-15 is available in the paper ‘Somatoform Disorders and Recent Diagnostic Controversies’^17^.

#### Mental health instruments

To assess the co-morbidity of depressive and anxiety symptoms, participants also completed the Patient Health Questionnaire (PHQ)-9 and Generalized Anxiety Disorder (GAD)-7 instruments^18^. A PHQ-9 score of 10 points or higher was used to estimate prevalence of DSM-IV major depressive episode^19^. PHQ-9 scores were reported as ‘depression symptoms’ in keeping with the established use as symptom severity measure. A GAD-7 score of 10 points or higher was used as the cut-off point for panic disorder, social anxiety and PTSD^12,20^. GAD-7 scores were reported as ‘anxiety symptoms’ in keeping with the established use as symptom severity measure.

#### Demographics

Sex (binary), age (categorical by three sub-groups: 18–30, 31–70 and more than 71 years) and socioeconomic status (categorical three-level variable: quintiles 1, 2–4 and 5). Socioeconomic status was classified by the Index of Relative Socioeconomic Advantage and Disadvantage (IRSAD) based on postcode of usual residence^21^.

#### Pre-admission healthcare utilization and costs

Pre-admission service use was determined by participant response to the healthcare utilization questionnaire, developed for the study by the author M.S. (*Fig. S1*). Costs were conservatively estimated from the Medicare Benefits Scheme (MBS) rebate^22^, which is a national standard for estimating inpatient and outpatient health costs, patient out of pocket expenses in addition to the baseline rebate were not included. All costs are reported in Euros, converted from Australian Dollars at the conversion rate as of February 2022. For 3 months before the index admission, participants reported the number of hospital presentations, general practitioner (GP) consultations (MBS Basic Consult AA010: €25.83), physiotherapy consultation (MBS Standard Consult PTA002: €52.82) and occupational therapy consultation (MBS Standard Consult 10958: €40.71). Imaging investigations costed included: ultrasound including urinary tract (OA095: €263.14), CT abdomen and pelvis with intravenous contrast (OD360: €1008.17), abdominal X-ray (58900 €81.16) and MRI abdomen (OP200: €1081).

#### Index admission healthcare utilization and costs

In-hospital service utilization costs for the index admission were conservatively estimated using the MBS funding schedule for public patients admitted to a subacute/non-acute bed. Duration of hospital stay was calculated from the clinical record and costed for an average (mean) subacute/non-acute bed at €901.02 per day^22^. Details were derived from the patients’ electronic medical record. Imaging costs were X-ray abdomen (€46.88), CT of abdomen and pelvis (€472.45), magnetic resonance cholecystopancreatogram (MRCP) (€255.66) and ultrasonography of the abdomen (55038 €107.40). ‘Blood panel’ was recorded as a single input, with value of full blood count (FBC: €10.75), electrolytes urea creatinine (EUC: €6.15), C-reactive protein (CRP: €6.15) and liver function tests (LFTs: €6.15), with a total value of €29.20 per patient per day. In our establishment most surgical patients will receive these tests on admission and once daily during admission as routine. In addition, any operative procedure in theatre (categorical) was recorded. An analysis of routine operations performed on the general surgery emergency operating list concluded that the average (mean) operating theatre use time was approximately 2 h per AGSU case (internal John Hunter Hospital audit). Hourly costing of theatre time was calculated using the New South Wales Government Agency for Clinical Innovation Operating Theatre Costs calculator^23^, and gave a total cost of €1268 per hour. Cost of attending theatre was thus totalled at an average (mean) of €2536 per patient. No participant returned to theatre during their index admission.

### Statistical analyses

Participant characteristics were stratified by PHQ-15 score of 10 or higher and disaggregated by sex, age group and socioeconomic status to report descriptive statistics (numbers and percentages within strata). Similarly, pre-admission and admission healthcare utilization were stratified by PHQ-15 scores of 10 or higher (reported as number and percentage for categorical variables and means and s.d.) for continuous variables). Differences between strata were tested using chi-squared or Student’s *t* tests as appropriate. *P* values were used to assess the strength of the evidence against the null hypothesis. Values between 0.1 and 0.05 were considered indicative of very weak evidence; values between 0.05 and 0.01 as weak evidence; values between 0.01 and 0.001 as moderate evidence, and less than 0.001 as strong evidence against the null hypothesis.

To compare the average (mean) costs of healthcare utilization by PHQ-15 strata, mean pre-admission costs (imaging, physiotherapy or occupational therapy consultation, and GP consultation costs), mean in-hospital costs (imaging, blood panel, subacute bed day and operating theatre) and mean total costs of healthcare were reported. Mean differences between the two strata (and 95 per cent c.i.) were estimated using univariable linear regression. The percentage difference (and 95 per cent c.i.) in costs between the two strata, calculated from the same linear regression model using semi-elasticities (the mean difference in log costs on the exponential scale) were also reported. The total healthcare utilization costs by PHQ-15 scores were examined using a multivariable linear regression. Along with PHQ-15 scores, the model included GAD-7 scores, PHQ-9 scores, theatre attendance, age group, sex and socioeconomic quintile as covariates. The coefficients were then used to estimate marginal mean costs at different PHQ-15 values. This was reported graphically for the relationship of total healthcare costs against PHQ-15 scores from the multivariable model.

As subacute bed day costs (duration of hospital stay) comprised the largest single component of total healthcare costs and the greatest cost difference, a post hoc exploratory analysis was conducted using a multivariable linear regression analysis to estimate the magnitude and direction of the association of variables expected might be associated with longer duration of hospital stay (1-day units). Predictors were PHQ-15 scores, GAD-7 scores, PHQ-9 scores, theatre attendance, sex, and age group. All analyses were conducted in Stata version 16.1 (StataCorp, College Station, Texas, USA). These data are not publicly available due to ethics restrictions.

## Results

A total of 731 patients were invited to participate in the study and 590 questionnaires were returned (80.7 per cent event participation rate). After removal of 96 incomplete data sets, and 29 repeat admissions in the study interval (ensuring that only index admissions were used), 465 records were available for analysis in this study (response rate 63.6 per cent of eligible admissions and 82.9 per cent of participating individuals).

### Participant characteristics

Participant characteristics are shown in *Table 1*. Of the 465 participants, sex was nearly equal (52 per cent female), 60 per cent were aged 31–70 years and 86 per cent from the three middle socioeconomic quintiles (Q2–Q4), with 52 per cent having medium to high somatic symptom severity (PHQ-15 score of 10 or higher).

**Table 1:** Participant characteristics stratified by PHQ-15 score cut-off point of 10 or higher

	PHQ-15 <10	PHQ-15 ≥10	Total
	223 (48.0)	242 (52.0)	465 (100)
**Sex**			
Female	102 (45.7)	143 (59.1)	245 (52.67)
Male	121 (54.3)	99 (40.9)	220 (47.31)
**Age (years)**			
17–30	45 (20.18)	53 (22.08)	98 (21.17)
31–70	140 (62.78)	138 (57.5)	278 (60.04)
≥71	38 (17.04)	49 (20.42)	87 (18.79)
**IRSAD**			
Quintile 1 (low)	21 (9.46)	27 (11.30)	48 (10.41)
Quintiles 2–4	191 (86.04)	207 (86.61)	398 (86.33)
Quintile 5 (high)	10 (4.50)	5 (2.09)	15 (3.25)

IRSAD, Index of Relative Socioeconomic Advantage and Disadvantage; PHQ, Patient Health Questionnaire. Values are *n* (%).

### Healthcare utilization stratified by PHQ-15 score cut-off point of 10 or higher

Participant healthcare utilization, stratified by PHQ-15 score of 10 or higher, is shown in *Table 2*. The group with a PHQ-15 score of 10 or higher had on average (mean) significantly more hospital presentations (1.2 *versus* 0.7), GP consultations (2.2 *versus* 1.4) and imaging investigations (1.7 *versus* 0.9), with no difference in the number of physiotherapy or occupational therapy consultations. Admission healthcare utilization is also shown in *Table 2*. The group with a PHQ-15 score of 10 or higher had on average (mean) significantly longer duration of hospital stay (5.1 *versus* 3.8 days) and more routine blood panels (4.6 *versus* 4), with no difference on imaging or theatre attendance.

**Table 2:** Healthcare utilization in the 3 months before admission and during hospital admission

	PHQ-15 <10 (*n* = 223)	PHQ-15 ≥10 (*n* = 242)	*P* value
**Pre-admission healthcare utilization**			
Hospital Presentations	0.7(1.1)	1.2(1.5)	<0.001
Imaging	0.9(1.8)	1.7(2.3)	<0.001
Physiotherapy or OT consultation	0.4(1.3)	0.5(1.6)	0.282
GP consultations	1.4(1.8)	2.2(2.2)	<0.001
**Admission healthcare utilization**			
Subacute bed days (LOS)	3.8(3.7)	5.1(5.1)	0.002
Routine blood panel	4(2.1)	4.6(2.1)	0.002
Imaging	1.8(1.6)	2.0(1.5)	0.149
Theatre attendance *n* (%)	105 (47.09%)	99 (40.91%)	0.180

PHQ, Patient Health Questionnaire; GP, general practitioner; OT, occupational therapist; LOS, length of stay. Values are mean(s.d).

### Healthcare costs stratified by PHQ-15 score cut-off point or 10 or higher

Participants mean pre-admission, admission and total costs, stratified by PHQ-15 score cut-off point of 10 or higher is shown in *Table 3*. The group with a PHQ-15 score of 10 or higher had significantly greater pre-admission healthcare costs (mean difference €391.86 and percentage increase 83 per cent), admission costs (mean difference €1010.08 and percentage increase 20 per cent) and total costs (mean difference €1401.93 and percentage increase 25 per cent).

**Table 3:** Healthcare costs in the 3 months before admission and during hospital admission

	PHQ-15 <10, mean	PHQ-15 ≥10, mean	Mean difference (95% c.i.)	Proportionate change (95% c.i.)	*P* value
**Pre-admission costs**					
Imaging	€421.02	€781.18	€360.15 (€174.37 to €545.94)	86% (30 to 166)	<0.001
Physiotherapy or OT consultation	€13.95	€24.09	€1.05 (€−1.90 to €22.83)	74% (−17 to 261)	0.104
GP consultation	€35.51	€57.07	€21.56 (€12.05 to €31.07)	61% (28 to 101)	<0.001
Total pre-admission	€470.48	€862.34	€391.86 (€202.90 to €581.44)	83% (32 to 155)	<0.001
**Admission costs**					
Subacute bed days (LOS)	€3427.15	€4568.48	€1141.96 (€405.81 to €1878.75)	33% (10 to 61)	0.002
Routine blood panel	€117.30	€135.06	€17.75(€6.34 to €29.17)	15% (5 to 26)	0.002
Imaging	€381.71	€421.66	€40.58 (€−20.92 to €101.45)	11% (−5 to 29)	0.194
Theatre attendance	€1251.02	€1058.26	€−192.76 (€−447.65 to €62.14)	−15% (−32 to 6)	0.138
Total admission	€5174.02	€6184.10	€1010.08 (€153.45 to €1866.07)	20% (3 to 39)	0.021
**Total cost of care**	€5644.50	€7046.43	€1401.93 (€512.19 to €2273.67)	25% (8 to 44)	0.002

PHQ, Patient Health Questionnaire; GP, general practitioner; OT, occupational therapist; LOS, length of stay (days).

The greatest cost difference for the pre-admission interval was for imaging investigation (mean difference €360.15 and percentage difference 86 per cent), and for the admission interval daily subacute bed costs (mean difference €1141.96 and percentage increase 33 per cent).

### Somatic symptom severity and total healthcare costs

**Fig. 1:**
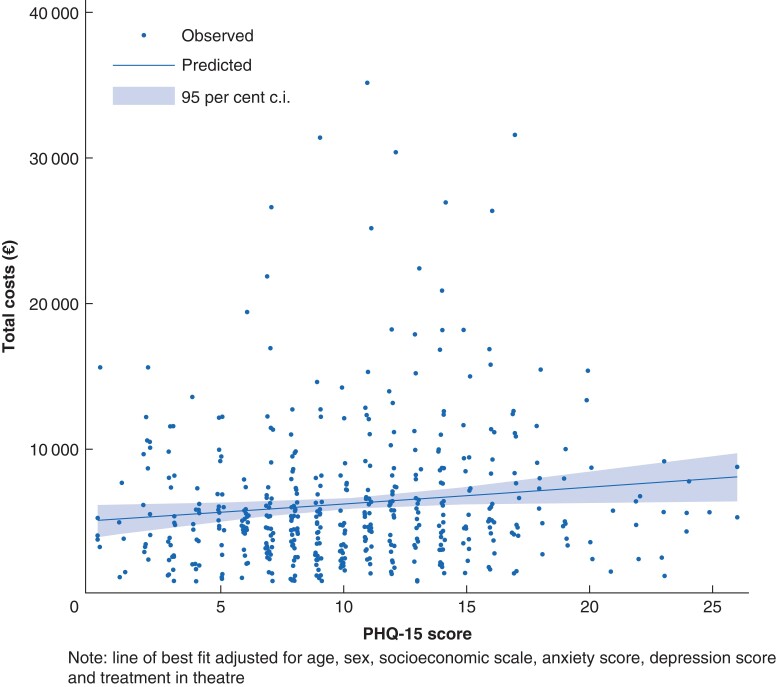
**Total costs of healthcare by PHQ-15 score (from model 1 in** ***Table 4*** **)**

In the multivariable model, PHQ-15 scores were associated with increasing total healthcare costs with each one-point increase in PHQ-15 scores was associated with a €116.04 increase in healthcare costs (95 per cent c.i. €13.32 to €218.12) (*Table 4*). The mean total cost of care was €1160.35 higher for a patient with a PHQ-15 score of 15 than for a patient with a score of 5 (*Fig. 1*).

PHQ, Patient Health Questionnaire.

**Table 4:** Multivariable predictors of total healthcare costs using linear regression

Predictor	Coefficient (95% c.i.)	Proportionate change (95% c.i.)	*P* value
PHQ-15 score*	115.21 (13.49 to 216.92)	2.1% (0.2 to 4.1)	0.027
GAD-7 score*	−77.43 (−191.84 to 36.95)	−1.4% (−3.6 to 0.7)	0.180
PHQ-9 score*	93.97 (−16.56 to 204.50)	1.7% (−0.3 to 3.8)	0.010
**Attended theatre**			<0.001
No (ref.)	—	—	
Yes	3758.529 (2948.97 to 4568.08)	63.8% (47.4 to 80.2)	
**Age group**			<0.001
17–30 years (ref.)	—	—	
31–70 years	1397.88 (398.24 to 2397.52)	30.6% (4.7 to 56.5)	
≥71 years	3552.87 (2256.52 to 4849.22)	63.3% (35.5 to 91.1)	
**Sex**			0.730
Male (ref.)	—	—	
Female	139.92 (−660.59 to 940.43)	2.6% (−12.3 to 17.5)	
**IRSAD**			0.064
Quintile 1	2689.14 (192.44 to 5185.83)	53.3% (−13.9 to 120.4)	
Quintiles 2–4	1430.30 (−780.46 to 3641.07)	32.6% (−32.6 to 97.7)	
Quintile 5 (high, ref.)	—	—	
***R* ^2^ statistic**	21.6%		

*Per one-point increase in PHQ-15, GAD-7 or PHQ-15 scores. PHQ, Patient Health Questionnaire; GAD, Generalized Anxiety Disorder; IRSAD, Index of Relative Socioeconomic Advantage and Disadvantage.

There were other predictors of total healthcare costs in the multivariable model. Each one-point increase in PHQ-9 (depression) scores was associated with a €94.90 increase, whereas GAD-7 (anxiety) scores showed no association. Theatre attendance, older age and lower socioeconomic disadvantage were significantly associated with increased total healthcare costs; however, sex had no association.

### Duration of hospital stay and somatic symptom severity

Recognizing that duration of hospital stay (daily subacute bed cost) was the single variable with the greatest cost differential, a post hoc multivariable linear regression analysis was performed to examine the association of PHQ-15 scores with duration of stay. PHQ-15 scores were not significantly associated with longer duration of stay; however, the magnitude of the effect and 95 per cent confidence interval (0.10 days; −0.001 to 0.20) for each one point on the PHQ-15 score showed a similar pattern to the results seen in the total healthcare multivariable model. Thus, a 10-point increase in PHQ-15 scores was associated with one extra day in hospital. Adjusted for covariates, a patient with a PHQ-15 score of 0 would be predicted to spend 3.4 days (on average (mean)) in hospital, whereas a patient with a score of 10 would spend 4.4 days in hospital and a patient with a score of 20 would spend 5.3 days in hospital. (*Table 5*)

**Table 5:** Multivariable predictors of inpatient duration of hospital stay (in days) using linear regression

Predictor	Coefficient (95% c.i.)	*P* value
**PHQ measures**		
PHQ-15 score*	0.10 (−0.001 to 0.20)	0.060
GAD-7 score*	−0.04 (−0.15 to 0.07)	0.482
PHQ-9 score*	0.06 (−0.04 to 0.17)	0.243
**Attended theatre**		<0.001
No (ref.)	—	
Yes	1.50 (0.70 to 2.30)	
**Age (years)**		<0.001
17–30 (ref.)	—	
31–70	1.26 (0.27 to 2.24)	
≥71	3.27 (1.99 to 4.54)	
**Sex**		0.859
Male (ref.)	—	
Female	−0.07 (−0.86 to 0.72)	
**IRSAD**		0.042
Quintile 1	2.74 (0.28 to 5.19)	
Quintiles 2–4	1.34 (−0.84 to 3.51)	
Quintile 5 (high, ref.)	—	
***R* ^2^ statistic**	9.7%	

*Per one-point increase in PHQ-15, GAD-7 or PHQ-15 scores. PHQ, Patient Health Questionnaire; GAD, Generalized Anxiety Disorder; IRSAD, Index of Relative Socioeconomic Advantage and Disadvantage.

## Discussion

This study demonstrated that greater somatic symptoms severity was associated with higher healthcare utilization and higher healthcare costs, whether considered as the medium to high classification (PHQ-15 cut-off point of 10 or higher) or as a continuous score. The cost differences for one episode of abdominal pain requiring admission were substantial; pre-admission (83 per cent higher), in-hospital (20 per cent higher) and total costs (25 per cent higher), which was considered clinically and economically significant. Somatic symptom severity as a continuous score was independently associated with higher total costs, even after adjustment for key predictor variables, including PHQ-9 and GAD-7 scores (psychological co-morbidity). Each one-point increase in PHQ-15 score was associated with a €116.04 increase in total costs, so that a patient with a PHQ-15 score of 15 would on average (mean) cost €1165 more per episode than a patient with a PHQ-15 score of 5. Our findings are generally consistent with some of the literature that has shown higher healthcare costs associated with somatization in primary care populations^8^.

### Surgical management implications

Duration of hospital stay was the single component of inpatient care that contributed most to the difference in health service costs. This is an observational study design, so it cannot be asserted that this association is causal; however, it can be speculated that there might be three groups of influences that might explain this association: patient factors, clinician factors (surgeons) and system factors.

Patient factors include the burden of somatic symptoms, as well as the complex nature of psychiatric illness. Increasing somatic symptom severity is associated with decreased functional status, symptom-related difficulty, and healthcare utilization^5^. A primary care study identified graphic emotional language, complex patterns of symptoms that resist explanation, and the emotional and social impact of the symptoms as causative factors leading to over-investigation of patients with medically unexplained symptoms^24^. Some patients suffering from somatoform syndromes may have a strong personal belief in an underlying organic illness, and often seek further diagnostic tests and therapies. This may lead to multiple ineffective therapeutic attempts, resulting in frustration of the patients, and a difficult doctor–patient relationship^25^.

Physician fear of missing underlying organic pathology has been recognized as a factor in under-diagnosis of somatoform syndromes, and may encourage longer admissions and repeat investigations^26^. Importantly, in our population, high PHQ-15 scores were not associated with increased rates of operative management, or significant increase in inpatient imaging investigations, and therefore these were not factors driving higher healthcare costs or longer duration of stay. Instead, surgeons may be using a ‘watch and wait’ approach to identify organic pathology and avoid iatrogenic harm.

In this tertiary care facility, acute surgical patients are handed over to the following on-call consultant every 24 h, with consultant-led ward rounds daily. While this process allows fresh eyes on complex patients, from senior clinicians, potentially eliminating confirmation bias, it also hinders the development of trust and rapport. In addition, serial examination by the same clinician, is highly sensitive for detecting incongruent or inconsistent physical findings (findings that do not fit a known physical pathology, or that change over time), which are sometimes features of somatoform syndromes^4^. There may be inadequate use of consultation by, and collaboration with mental health clinicians available in the general hospital, which might be of assistance for some patients.

This model identified several independent predictors of total healthcare costs in addition to PHQ-15 scores; age, socioeconomic status, operative procedure in theatre, and PHQ-9 scores. Previous research suggests that older age^27^, socioeconomic status^28^ and psychiatric co-morbidity^29^ are all independently associated with longer and more complicated hospital admissions and are plausible predictors for increased duration of hospital stay in surgical inpatients.

Appropriate recognition for somatization is important, as it is amenable to change with evidence-based interventions. Although collaborative psychiatric consultation effectively improves functioning and reduces healthcare use for somatic symptom patients^30^, many patients are neither referred to psychotherapy by their primary care physician, nor do they initiate psychotherapy themselves^31^. Collaborative care trials, connecting primary care physicians, psychotherapists, and mental health clinics have shown feasibility, improved doctor–patient relationships, prescribing practices^32^, social functioning, and reduced medically unexplained symptoms^33^. In addition to collaborative care, systematic reviews have identified effective treatments for somatoform syndromes that improved patient outcomes and reduced health service costs, including cognitive behavioural therapy and antidepressants^34^, and so improved case identification and access to these interventions is warranted.

This study estimated the health service use and economic impact associated with somatic symptom severity in surgical inpatients, on a consecutive adult surgical inpatient population. A strength of this study is the broad inclusion criteria and a relatively large sample size. In addition, treating teams (surgeons) were kept blinded to the results of the surveys, so these could not influence their diagnostic and treatment decisions. A limitation of the study is the healthcare utilization questionnaire developed by the author M.S. This non-validated self-reported questionnaire was used to record pre-admission healthcare utilization, which was reliant on participant recollection. Although non-validated, the responses of the patients were tested by a comprehensive chart review for a representative sample of the cohort, which showed that the patients’ recollection of their attendance at hospital, and their use of hospital-based tests, was very accurate^35^. The MBS was used to estimate health expenditure, as this is the national standard in Australia; however, this is not comparable to Diagnosis Related Groups or Casemix used in other countries, and thus international comparisons must be made with caution. Another limitation was the recruitment processes, which may have missed some very brief overnight admissions or patients who were very unwell, especially those needing the ICU. A minority of eligible patients did not consent to the study and so their pattern of somatic symptoms and health service utilization is not known, and we did not have ethical clearance to record demographic data to compare the two populations. In addition, the assessment tool used to screen for somatic symptoms while admitted to a secondary care facility for investigation of (predominantly) abdominal pain itself includes abdominal pain as one of the items scored. This may lead to overestimation of the prevalence of somatoform syndromes. Our study population was limited to surgical inpatients with abdominal pain, and generalization of our results to other clinical populations (such as gastroenterology) should be performed with caution. There are legitimate questions about the use of questionnaires alone, to establish the prevalence of somatization. These include that the sensitive nature of psychiatric questionnaires may lead to response bias. In addition, the validity of using the PHQ-15 score in an older population is questionable^20,36^. Structured interviews or psychiatrist assessments were not used to produce estimates of any specific somatic syndrome. In addition to these issues, the study was restricted to a single episode of inpatient care and did not estimate long-term or lifetime prevalence of symptoms nor estimate quality of life or function in these patients.

Collaborative multidisciplinary teams can improve communication and evidenced-based patient care can reduce inappropriate service use, excess service costs^34^ and possibly reduce iatrogenic harm^33^. The results suggest a potential need for further integrated psychological assessment and intervention, which is not routinely offered during surgical inpatient admission.

## Funding

M.S. was funded by the University of Newcastle HDR Student Support Funding. M.J.S. was funded by an Australian Research Council Future Fellowship (project number FT180100075) funded by the Australian Government.

## Supplementary Material

zrac046_Supplementary_Data
