# Supplementary figures and images for: Somatic symptom severity association with healthcare utilization and costs in surgical inpatients with an episode of abdominal pain
Source: BJS Open. 2022 Jul 7;6(4):zrac046. doi: 10.1093/bjsopen/zrac046 (PMC9260183; doi:10.1093/bjsopen/zrac046)

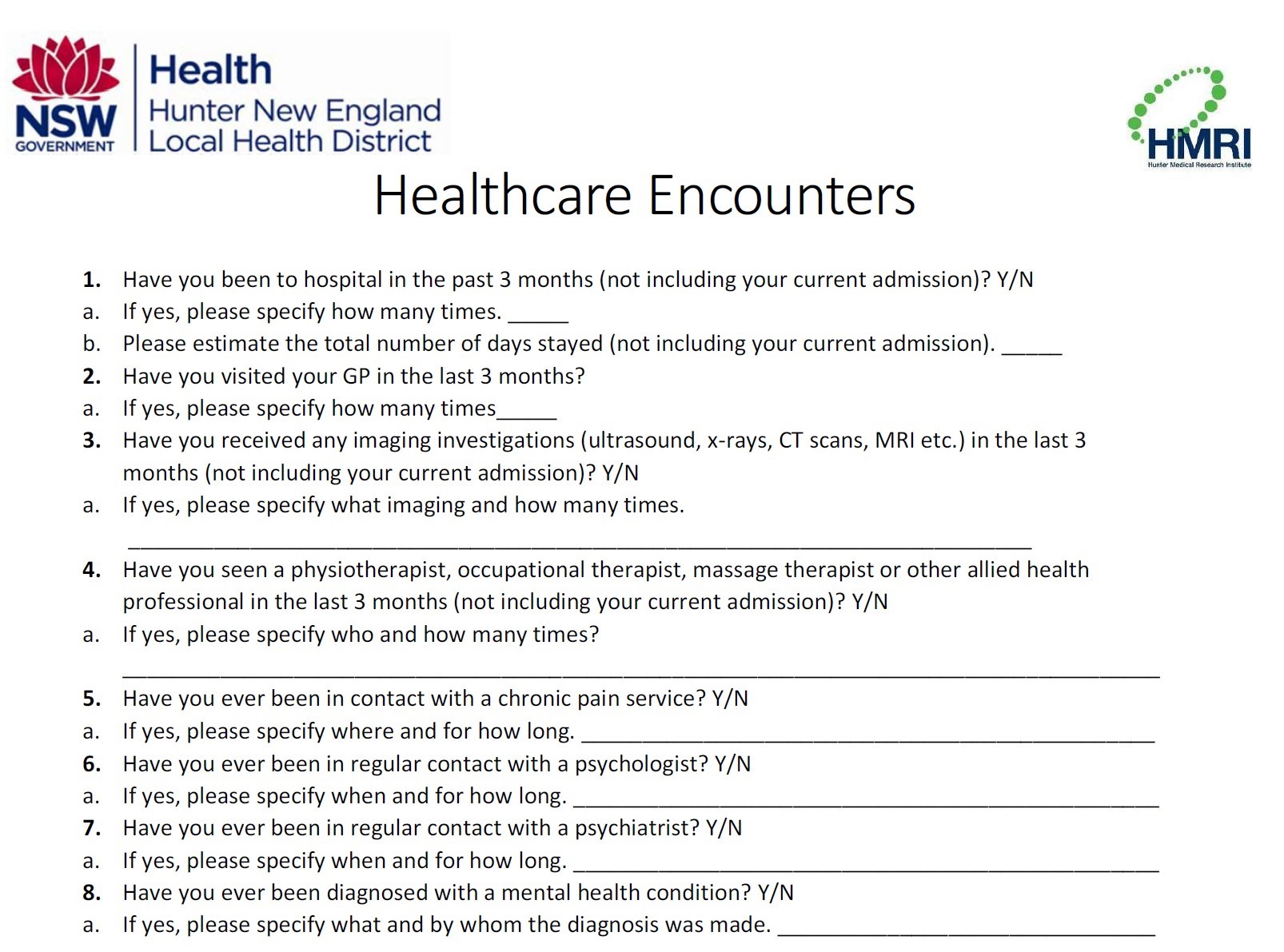

Supplement: zrac046_Supplementary_Data [file zrac046_supplementary_data.jpeg]
